# Retrograde Positive Contrast Urethrocystography of the Fish Urogenital System

**DOI:** 10.1155/2014/384165

**Published:** 2014-08-12

**Authors:** Francesco Macrì, Annamaria Passantino, Michela Pugliese, Simona Di Pietro, Daniele Zaccone, Pietro Giorgianni, Rossella Bonfiglio, Fabio Marino

**Affiliations:** ^1^Department of Veterinary Sciences, University of Messina, Polo Universitario dell'Annunziata, 98168 Messina, Italy; ^2^Department of Animal Biology and Marine Ecology, Faculty of Science, University of Messina, 98166 Messina, Italy

## Abstract

The radiological differences between the urinary tract of  *Dicentrarchus labrax*, *Sparus aurata*, *Tinca tinca*, and *Cyprinus carpio* are shown. In fresh water teleosts the urinary bladder is sigmoid and a short urethra leads to the urinary pore. Genital and anal pores are present. In *Sparus aurata* the urinary bladder has a globoid shape. In *Dicentrarchus labrax* the urinary bladder is smaller and elongate. In both marine teleosts a single urogenital pore is visible. Positive contrast was used to survey the urogenital system and evaluate shape and size of the bladder, urethra, ureter, and gonadal ducts. Results demonstrate the morphological variability of the urinary bladder and the craniodorsal entry of the ureters into the bladder. It is envisaged that this work will provide baseline information for further imaging studies for investigating the urogenital morphology and can be applied to identify disorders in fishes. Furthermore, the main interest of this study is that it demonstrates the morphological variability of the lower urinary system that exists between different species of fishes.

## 1. Introduction

Many teleosts have a urinary bladderwhich may take the form of a simple dilatation of the ureter or a saccular evagination. It usually is very small, its volume ranging from 0.4 to 0.7 mL per 100 g body weight. The urinary bladder of teleosts is an accessory osmoregulatory organ to the kidney, where urine is retained for some time. The portion of the ureter which carries urine from the bladder to the outside is called the urethra [1]. In freshwater fish, the urinary bladder is an important location for Na^+^, Cl^−^, and K^+^ (with a minimum of water) uptake from urine. However, in seawater teleosts the bladder does not only reabsorb Na^+^, Cl^−^, and water from urine but also concentrates urine excretion of particularly Ca^2+^, Mg^2+^, and SO_4_^2−^ [2].

Freshwater teleost fish excrete relatively large volumes of dilute urine (around 35 mOsmol/L) due to a high glomerular filtration rate and to an almost complete reabsorption of NaCl. Ions remaining in the tubules are further absorbed by the urinary bladder. Marine teleosts generally only produce low volumes of urine due to a low glomerular filtration rate; the kidney of the marine teleost is unable to concentrate salts in the urine and the fluid extracted is still hypoosmotic compared to plasma and contains relatively low concentrations of NaCl [3].

Gonadal ducts are present in most species to carry the gametes to their appropriate internal or external destinations. The basic organization of the gonads is similar in most vertebrates; however, structural variations among species occur, reflecting phyletic patterns or species-specific adaptations to the environment [4].

Radiology has today achieved an important role in clinical and morphological evaluation of many teleosts species [5, 6].

The application of radiology to teleosts has been reported [7–16].

The aim of this study is to analyze contrast media applications to assess the urinary bladder, ureters, gonadal ducts, and urethra in four species of freshwater and sea water teleosts.

Retrograde positive contrast urethrocystography is commonly used in small animal veterinary medicine to assess urinary tract integrity after a trauma and rule out a possible urinary bladder/urethral/ureteral rupture, or to assess a potential urethral obstruction due to an intraluminal (calculi, mucus plugs, and blood clot), mural (neoplasia, urethral stricture), or extramural lesion.

## 2. Materials and Methods

Contrast radiography (retrograde positive contrast urethrocystography) was carried out on 20 subjects: two fresh water species, tench (*Tinca tinca*, Linnaeus 1758) (*n* = 3) (230 g and 30 cm long (total length)) and carp (*Cyprinus carpio*, Linnaeus 1758) (*n* = 3) (450 g and 32 cm long (total length)), and two marine teleosts, seabass (*Dicentrarchus labrax*, Linnaeus 1758) (*n* = 3) (89 g and 19 cm long (total length)) and gilthead seabream (*Sparus aurata*, Linnaeus 1758) (*n* = 3) (90 g and 20 cm long (total length)). Fish were reared in 500 L experimental tanks at the CISS (Sicilian Centre for Experimental Fish Pathology) of the University of Messina.

The fish were anesthetized by placing them in a water tank containing tricaine methanesulfonate (MS-222; Sandoz, Switzerland, Le Locle-Neuchâtel) 0.3 mg L^−1^ for 3 min (marine teleosts) or 5 min (fresh water teleosts); water temperature was 24°C (fresh water) and 20°C (marine water). All fish were sufficiently sedated when initially placed on the X-Ray Cassette. Contrast medium was placed in the urinary system by inserting an intravenous catheter (20 gauges, 1.0 × 32 mm) into the urinary pore. A 2.5 mL syringe filled with nonionic-iodinated water-soluble contrast medium (iopamidol; Iopamiro 300 mg mL^−1^; Bracco Imaging Italia S.r.l., Milano) was used to fill the system. Injection ceased as soon contrast medium began to leak from the pore, resulting in a dose between 0.4 and 1 mL. After injection, radiography was performed by a Univet LX 160, Supply 230 VAC, 50 Hz, 6 kVolts, Power 99 kVolts, 160 mA. Because most specimens were less than ten centimeters thick, a grid was not considered necessary because the risk of compromising the quality of the image was low. High definition intensifying screens and detail film were used to optimize radiographic detail. Kodak T-MAT G/RA film in a X-Ray cassette was used.

All fish were radiographed in two projections: dorsoventral and right lateral. Lateral radiographs were obtained using exposure settings of* T. tinca,* 43 kV and 6.3 mAs;* D. labrax* 40 kV and 6.3 mAs;* C. carpio* 50 kV and 6.3 mAs;* S. aurata* 40 kV and 6.3 mAs. Dorsoventral radiographs were obtained using exposure settings of* T. tinca,* 47 kV and 6.3 mAs;* D. labrax* 44 kV and 6.3 mAs;* C. carpio* 54 kV and 6.3 mAs;* S. aurata* 44 kV and 6.3 mAs.

To maintain correct positioning, sandbags were used; this is an acceptable practice provided that there is no obstruction of anatomical structures in dorsoventral projections. In performing the lateral and dorsoventral projections, the X-ray beam was centered on the abdominal area. After radiography, the fish were placed in a tank with clean water, with the same water parameters as above described, until complete recovery was achieved (about 20 m). No side effects were registered during and after the study. Three months after this imaging investigation fish were alive and showed normal behavior.

## 3. Results

**Figure 1:**
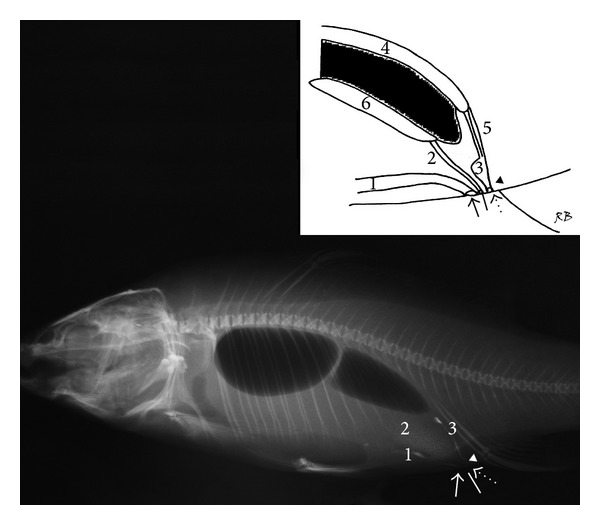
*Cyprinus carpio*: right lateral radiograph; inset: diagram of the urogenital system of* C. carpio*: anal pore (small arrow), genital pore (white line), urinary pore (dashed arrow), urethra (arrow head), (1) intestine, (2) gonadal duct, (3) empty urinary bladder, (4) kidney, (5) ureters, and (6) gonads.

**Figure 2:**
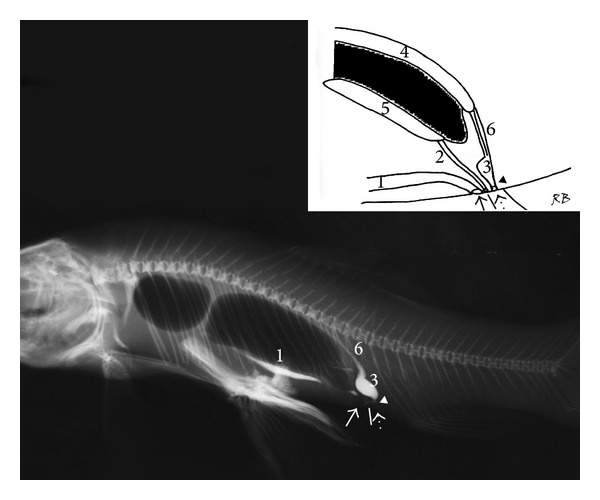
*Tinca tinca*: right lateral radiograph; inset: diagram of the urogenital system of* T. tinca*: anal pore (small arrow), genital pore (white line), urinary pore (dashed arrow), urethra (arrow head), (1) intestine, (2) gonadal ducts, (3) urinary bladder, (4) kidney, (5) gonads, and (6) ureters.

The retrograde positive contrast urethrocystography revealed the morphology of the urogenital apparatus in specimens. The urinary bladder in the* T. tinca *and the* C. carpio* showed a sigmoid shape in the lateral projection, length 1.2 to 1.5 cm (mean 1.3 cm), height 0.5 to 0.8 cm (mean 0.7 cm), and width (measured in dorsoventral projection) 0.5 to 0.7 cm (mean 0.6 cm). A short urethra connects the bladder to the urinary pore; the genital and anal pores were observed cranial to the urinary pore. The genital pore was clearly observed only in a mature* C. carpio* because of the presence of eggs within the lumen; in the other* C. carpio*, it was very small and virtually closed, whereas the enteric tube was observed cranial to the genital pore (Figures 1 and 2).

**Figure 3:**
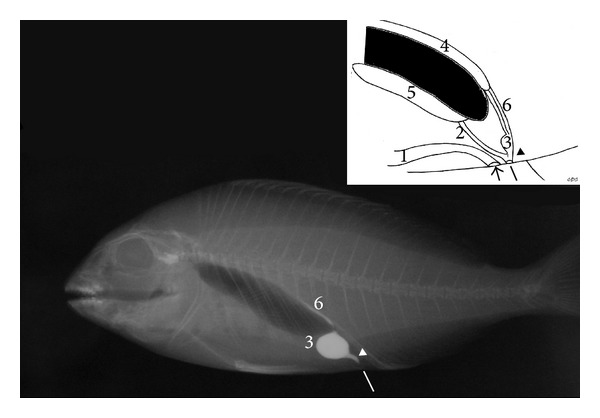
*Sparus aurata*: right lateral radiograph; inset: diagram of the urogenital system of* S. aurata*: anal pore (small arrow), urogenital pore (white line), urethra (arrow head), (1) intestine, (2) gonadal duct, (3) urinary bladder, (4) kidney, (5) gonads, (6) ureters.

**Figure 4:**
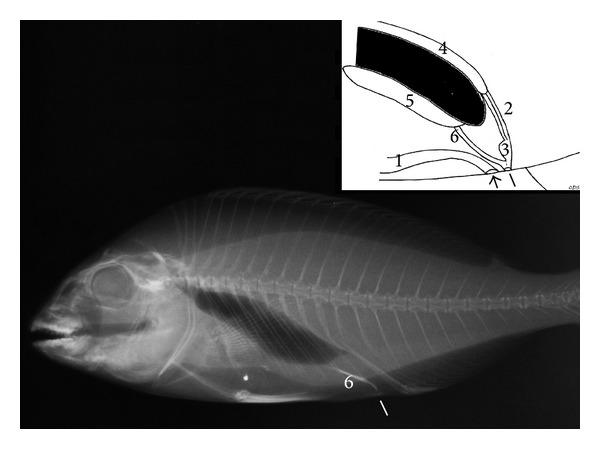
*Sparus aurata*: right lateral radiographic; inset: diagram of the urogenital system of* S. aurata* anal pore (small arrow), urogenital pore (white line), (1) intestine, (2) ureters, (3) urinary bladder, (4) kidney, (5) gonads, and (6) gonadal duct.

In* S. aurata*, the urinary bladder has a globoid shape, length 1.0 to 1.3 cm (mean 1.2 cm), height 0.8 to 1.1 cm (mean 0.9 cm), and width 0.1 to 0.3 cm (mean 0.2 cm) (Figure 3). The genital duct merged into the terminal portion of the urethra, forming a single urogenital pore (Figure 4).

**Figure 5:**
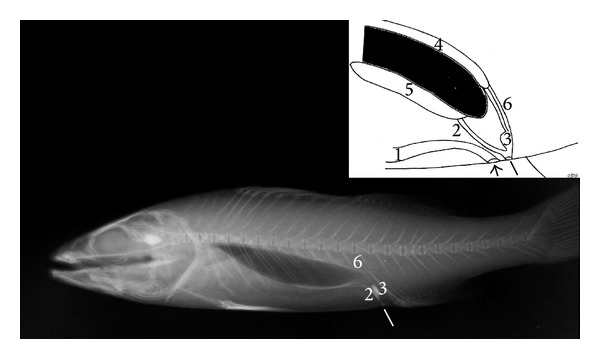
*Dicentrarchus labrax*: right lateral radiograph; inset: diagram of the urogenital system of* D. labrax*: anal pore (small arrow), urogenital pore (white line), (1) intestine, (2) gonadal duct, (3) urinary bladder, (4) kidney, (5) gonads, and (6) ureters.

In the* D. labrax*, the bladder is smaller and shows a thin and elongated shape resembling a rice kernel. The length is 0.4 to 0.6 cm (mean 0.5 cm), height 0.1 to 0.3 cm (mean 0.2 cm), and width 0.1 to 0.3 cm (mean 0.2 cm). Infusion of contrast medium through the urogenital pore permitted visualisation of both the urethra and genital duct. The genital duct is located ventrally to the urinary system and, in a lateral projection, its course is easily detectable until the area of the gonads (Figure 5).

In all the studied specimens, two ureters were seen to merge craniodorsally into the urinary bladder. They were easily filled by the contrast medium because of the lack of ureteral papillae as compared to the higher vertebrates, including humans.

## 4. Discussion

Positive urogenital contrast in fish is an excellent method to determine the location and to evaluate the shape and size of the urinary bladder, urethra, ureters, and gonadal ducts.

The three-dimensional arrangement of the urinary tract was reported for all four species examined here, and the arrangement of the ureters, which end in the dorsocranial portion of the bladder, was documented. This is in contrast to most mammalians, where they enter the urinary bladder dorsocaudally. However as in mammals, the urinary bladder drains into a single urethra exiting to the surface.

The tench (*T. tinca*) and common carp (*C. carpio*) are both freshwater and brackish water fish of the cyprinid family, living in slow-moving freshwater habitats, particularly lakes and lowland rivers [17]. It has been reported that in these species, as in most cyprinids, there are two macroscopically discernable separate kidneys; collecting ducts lead into the ureters and each ureter leads into the common cloaca, via a rudimentary urinary bladder [18]. In* C. carpio* and* T. tinca*, the genital ducts terminate at the genital pore, which opens between the anal pore cranially and the urinary pore caudally.

*D. labrax*, a member of the Moronidae family, is a primarily ocean-going fish that sometimes enters brackish and fresh water. Its habitats include estuaries, lagoons, coastal waters, and rivers.* D. labrax *are euryhaline fish that tolerate wide salinity fluctuations owing to several morphofunctional adaptations. Among the osmoregulatory sites (tegument, branchial chambers, digestive tract, and urinary system), little is known about the kidney and the urinary bladder.

The* S. aurata* is a euryhaline teleost able to control water and electrolyte content through different mechanisms of hyper- and hypoosmoregulation according to the external salinity. In both the marine species studied the genital duct and urinary duct exit as two separate ducts into two separate pores.

The different osmoregulatory function of the urinary bladder in fresh water fish, as compared to marine ones, let us suppose a different size in the urinary bladders of these two groups: fresh water fish should have a greater urinary bladder as compared to marine fish. On the contrary, our results do not underline this difference.

Radiographic exam demonstrated that the marine water fish species here studied showed urinary bladders with extremely different sizes; particularly* D. labrax* and* S. aurata*, both marine fish, had sharply different features.

It is envisaged that this work will provide baseline information for further imaging studies for investigating the urogenital morphology and can be applied to identify disorders in fishes. Furthermore, the main interest of this study is that it demonstrates the morphological variability of the lower urinary system that exists between different species of fishes.
